# The effect of Z-primer on the shear bond strength of zirconia ceramic to dentin: *in vitro*

**DOI:** 10.4317/jced.54619

**Published:** 2018-07-01

**Authors:** Anahit Afrasiabi, Elahe Mostajir, Niusha Golbari

**Affiliations:** 1 Resident of Operative Dentistry, Shahid Beheshti University of Medical Science, Tehran, Iran

## Abstract

**Background:**

One of the major limitations of zirconia ceramic is the difficulty to adhere to dental. Said adhesion improves the marginal sealing of the restoration, minimizing microleakage. According to the limitation of zirconia ceramic bonding, zirconia primer enhances bond strengths to Zirconia, Alumina and Metal substrates. Purpose: the aim of the current study was to determine effect of Z-primer on the shear bond strength of zirconia ceramic to dentin.

**Material and Methods:**

28 newly extracted intact premolars and divided into 4 groups (n=7). In group 1, resin-based (Duo-link; Bisco, Schaumburg, IL) cement was used without applying Z-primer. In group 2, resin-based (Duo-link; Bisco, Schaumburg, IL) cement was used with applying Z-primer. In group 3, resin-based cement (Panavia F2; Kurary, Japan) was used without Z-primer. In group 4, resin- based cement (Panavia F2) with Z-primer was used. Zirconia ceramic blocks (4×4×2mm) without sandblasting were applied onto the surface of dentin then cured from 3 dimensions for 20S. Shear bond strength test was carried out after 24h. Stereo microscope was used to evaluate the zirconia ceramic and dentin topography and failure mode.

**Results:**

According to the data, significant difference detected on after application of the Z-primer compared to the other groups (*p*<0.001). No significant difference detected between 2 types of resin cements (*p*>0.05). However, the highest SBS observed in second group (Duo-link(+)Zprimer) while lowest detected in first group (Duo-link(-)Zprimer).

**Conclusions:**

These results suggested application of the Z-primer increased shear bond strength between zirconia ceramic and dentin.

** Key words:**Flexural strength, monolithic zirconia, sintering temperature, sintered-holding time.

## Introduction

The use of zirconia as a core material suggested because of its high chemical stability, modulus of elasticity, strength and esthetic properties (1). Zirconia has emerged as a versatile and promising ceramic material because of its biological, mechanical and optical properties. With a flexural strength of more than 900 MPa, fracture toughness of up to 10 MPa and an elastic modulus of 210 GPa, they exhibit better mechanical performance, superior strength and fracture resistance than do other ceramic materials (2). More than 50% incidence of veneering ceramic fracture on a zirconia substructure, especially in posterior teeth was reported (3). Adhesion of resin cement to high-strength zirconia ceramics is not expected to be improved by acid etching and silanization because they are inert acid resistant ceramics (2). For zirconia ceramics, airborne-particle abrasion is an alternative method for roughening the ceramic surface (4,5).

Bond strength in turn is affected by pretreatment procedures, the depth of cure and degree of polymerization of the resin cement, and incompatibilities between the adhesive resin and the resin cement. Factors that may affect polymerization include cement film thickness, opacity and translucency of both the cement and restoration and shade of the restoration (6). Resin cements and primers containing the acidic monomer 10-metacryloxydecyl dihydrogen phosphate (MDP) are the recommended cements for zirconia ceramics as MDP can chemically bond with zirconia (7). Examples of such cements and primers are Panavia F 2.0, SE Bond, SA Luting Cement (Kuraray, Osaka, Japan) and the newer Scotchbond Universal adhesive (3 M Espe, Germany). Also, Z-Primer (Bisco) and AZ Primer (Shofu) which contains phosphoric acid monomers can also be used to promote the adhesion of alumina and zirconia due to chemical bond formation (8). It can be claimed that Z-primer increases bond strength to zirconia, Alumina and metal, because of MDP (Methacryloxydecyl Dihydrogen Phosphate) and BPDM (Biphenyl Demethacrylate) (9). It is reported zirconia primer based on organophosphate/carboxylic acid monomers increased the bond strength of zirconia posts to root canal dentin bonded with both resin luting cements (10). Also, the application of MDP-containing primer resulted in increased bond strength between Y-TZP ceramics and MDP-containing self-adhesive resin cements (11). Because of the high stability, the zirconia ceramic does not easily bond to the resin. Therefore, it is necessary to enhance bond strength, unlike the case where bonding occurs between the bracket and teeth (12). So, the aim of the current study was to determine effect of Z-primer on the shear bond strength of zirconia ceramic to dentin.

## Material and Methods

28 newly extracted intact premolars and divided into 4 groups (n=7). The teeth were cut from 1mm under the central groove to expose the dentin then they were embedded in acrylic resin base. The teeth were etched with phosphoric acid and had been applied by two layers of single Bond, then were cured. In group 1, resin-based (Duo-link; Bisco, Schaumburg, IL) cement was used without applying Z-primer. In group 2, resin-based (Duo-link; Bisco, Schaumburg, IL) cement was used with applying Z-primer. In group 3, resin-based cement (Panavia F2; Kurary, Japan) was used without Z-primer. In group 4, resin- based cement (Panavia F2) with Z-primer was used. Zirconia ceramic blocks (4×4×2mm) without sandblasting were applied onto the surface of dentin then cured from 3 dimensions for 20S. Shear bond strength test was carried out after 24h storage in water. Stereo microscope was used to evaluate the zirconia ceramic and dentin topography and failure mode.

-Statistical analysis

Data analyzed by repeated measure two-way analysis of variance (ANOVA) using SPSS 16.0 for Windows (SPSS, Inc., Chicago, IL, USA). For treatment showing a main effect by ANOVA, means compared by Tukey–Kramer test. *P*<0.05 was considered as significant differences between treatments.

## Results

As seen in  Table 1, the highest Shear Bond (Mpa) was observed in Duo-link (+) Z-primer (6.1757 Mpa). The groups PanaviaF2 (+) Z primer and PanaviaF2 (-) Z primer had 5.5916 and 4.1780 Mpa Shear Bond strength. The lowest Shear Bond strength detected in group 1 (Duo-link (-) Z-primer).

**Table 1:**
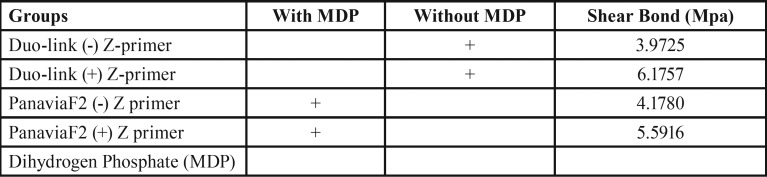
The Shear Bond (Mpa) of different cements with or without Z-primer.

As seen in  Table 2, there is no significant difference between the cements with or without MDP and also between their types (*p*>0.05).

**Table 2:**
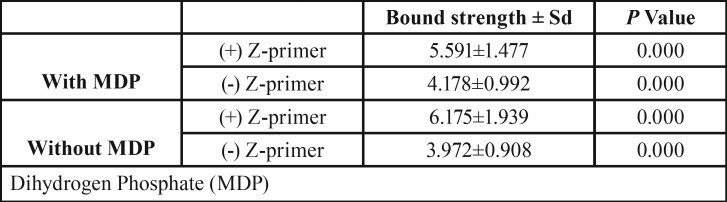
The comparison Shear bound strength of cements with or without Z-primer.

Significant difference detected between Shear bound strength of Z-primer with or without cements (*P*<0.001).

The samples were studied under stereo microscope with 20x magnification for failure mode evaluation. The failure mode was adhesive for the first group and predominantly cohesive for the other groups.

## Discussion

Developments over the last decades in ceramic materials science for dental applications have led to zirconia-based ceramics which potentially provide better fracture resistance and long-term viability compared to porcelain and other inorganic, non-metallic alternatives (13). Although superior in terms of mechanical performance such as strength, toughness and fatigue resistance, there are some inherent problems associated with zirconia (14). The bond strength between adherent materials can be tested using various test methods .The most common test methods for evaluating the adhesive bond strength is the shear bond strength test which has advantage of the easy to use (15).

The MDP was originally designed to bond to metal oxides and its use has been extended to oxide ceramics (16). MDP-containing resin cements seem to be the most appropriate due to the chemical interaction between the hydroxyl groups of the passive zirconia surface and the phosphate ester group of the MDP. Suggestions have been made that a chemical bond might be established between MDP and zirconia (14).

According to the data, significant difference detected on after application of the Z-primer compared to the other groups. No significant difference detected between 2 types of resin cements. However, the highest shear bond strength observed in second group (Duo-link (+) Z-primer) while lowest detected in first group (Duo-link (-) Z-primer). (18)reported Z-Prime Plus increase the bond strength of different resin luting agents (Biscem, Duo-link, Panavia F) (17). The association of the experimental primer with the restorative composite resin Z100 yielded the highest shear bond strength followed by Duo-Link with the new primer. The excellent shear bond strength obtained with Z-primer and Z100 also suggests that the new primer could be used for intraoral repair of chipped/failed restorations with exposed zirconia substructure. Duo-Link was used in order to match the Z-primer with a conventional resin luting agent from the same manufacturer. The resin parts of both Z100 and Duo-Link are mainly composed by methacrylate-based monomers, such as Bis-GMA which interacts directly with the organofunctional part of the Z-primer (18). The MDP is also present in Panavia F and the phosphate ester group of this monomer bonds chemically to aluminum and zirconium oxides (18). According to the literature, the presence of MDP in the resin luting agent creates stable bond strength to airborne particle abraded zirconia before and after thermo-cycling (19). The highest shear bond strengths values were reported with application of Signum Zirconia bond and. It is reported Signum Zirconia bond is effective for increasing the bond strength of adhesive resin cement to zirconia ceramic (20). It is demonstrated that application of an MDP-containing bonding/silane-coupling agent mixture to zirconium dioxide ceramic restorations abraded with airborne Al2O3 particles can yield superior shear bond strength. These results may be explained by the improved wettability of a rough zirconia surface due to the mixture of the bonding/silane agent and the presence of chemical bonding (21). According to the manufacturers, both the zirconia primers and the universal adhesives employ MDP as the main functional component for providing chemical bonding to Y-TZP. The phosphate monomer MDP was developed to achieve direct bi functional adhesion with metal oxides (including zirconia and alumina) and the bis-GMA matrix (22).

Many other innovative attempts have been developed to increase the resin-zirconia bond strength. Modification of the zirconia surface by sintering a porous ceramic layer on the milled zirconia allowing resin infiltration (NobelBond; Nobel Biocare AB, Goteborg, Sweden), transformation of zirconia into an etch able substrate by the selective infiltration technique (23). The clinical implication of this study is that the application of the Z-primer increased shear bond strength between zirconia ceramic and dentin.

## Conclusions

Shear bond strength value of zirconia porcelain to dentin is increased by using Z-primer. Studies on effect of resin cement with or without MDP showed no significant difference statistically. However, based on the acquired figures from the shear band strength test, maximum value was in the group of Duolink cement without MDP but including Z-primer and minimum was in the group of Duolink without applying Z-primer.
